# Cardiomyopathy in Africa: heredity versus environment

**Published:** 2007-07

**Authors:** Bongani M Mayosi, Krishna Somers

**Affiliations:** Department of Medicine, University of Cape Town and Groote Schuur Hospital, Cape Town; Department of Internal Medicine, Royal Perth Hospital, Perth, Australia

## Abstract

**Summary:**

Unlike other parts of the world in which cardiomyopathy is rare, heart muscle disease is endemic in Africa. The major forms of cardiomyopathy in Africa are dilated cardiomyopathy (DCM) and endomyocardial fibrosis (EMF). Whereas DCM is a major cause of heart failure throughout the continent, EMF is restricted to the tropical regions of East, Central, and West Africa. Although epidemiological studies are lacking, hypertrophic cardiomyopathy and arrhythmogenic right ventricular cardiomyopathy seem to have characteristics similar to those of other populations elsewhere in the world.

Recent advances in the genetic analysis of DCM in other parts of the world indicate that it is a genetically heterogeneous disorder in which some cases have a Mendelian cause and others have a non-genetic or multifactorial cause. This heterogeneous pattern of inheritance has been confirmed in small studies that have been conducted so far in Africa. The advent of human immunodeficiency virus infection and its association with cardiomyopathy has emphasised the role of inflammatory agents in the pathogenesis of DCM.

By contrast with DCM in which some cases have major genetic contributions, there is scanty evidence for the role of genetic factors in the aetiology of EMF. Although the pathogenesis of EMF is not fully understood, it appears that the conditioning factor may be geography (in its widest sense, to include climate and socio-economic status), the triggering factor may be an as yet unidentified infective agent, and the perpetuating factor may be eosinophilia. There is a need for renewed effort to identify genetic and non-genetic factors in EMF and other forms of heart muscle disease that are prevalent on the continent of Africa.

## Heredity vs environment in common disease

The sequencing of the human genome has raised afresh the age-old debate on the relative importance of nature (ie, heredity) and nurture (ie, environment) in the aetiology of human disease. There are, however, at least three lines of epidemiological evidence that emphasise the dominant role of environmental factors in the causation of the common forms of heart disease.

First, it is well established that the type and the burden of heart disease change over time as a country undergoes economic development. Developing countries begin with a disease profile that is dominated by nutritional, perinatal and infectious diseases and, in the process of development, make the transition to one dominated by non-communicable diseases, such as cardiovascular disease and cancer.^1^,^2^ Second, risk-factor interventions in populations living in industrialised countries have been associated with large reductions in cardiovascular mortality over the past 30 years. These dramatic shifts in the pattern of disease within a few generations in populations of the same genetic stock highlight the dominant role of environmental factors in the causation of common forms of heart disease, with hereditary factors playing only a minor role.

Indeed, the modest effect of inherited factors has been quantified at an epidemiological level in INTERHEART, the case-control study of risk factors for myocardial infarction conducted in all major continental populations, which has shown that family history (a combined measure of inherited factors) probably accounts for 1% of the overall population-attributable risk of the myocardial infarction.^3^ Specific genetic variants that increase the risk of cardiovascular disease have been discovered (eg, IL6 gene and carotid atherosclerosis),^4^ but in general, their effect on overall susceptibility to cardiovascular disease is small.^5^

## Cardiomyopathy: a common disease in Africa

Cardiomyopathy of undetermined cause has been known to be endemic in Africa for over 60 years.^6^ The majority of countries in Africa are still in the early stages of the epidemiologic transition, such that the predominant circulatory diseases are heart muscle disorders (cardiomyopathy), rheumatic valve disease, pericardial tuberculosis and cor pulmonale following pulmonary tuberculosis.^6^,^7^

The major forms of cardiomyopathy in Africa are dilated cardiomyopathy (DCM) and endomyocardial fibrosis (EMF).^6^ DCM accounts for 10 to 17% of cardiac conditions encountered at autopsy, and for 17 to 48% of patients who are hospitalised for heart failure. DCM is a disease that is found in all age groups and in all the regions of Africa. By contrast, EMF is a disease of children and young adults that is confined to the tropical regions of equatorial Africa. Undiagnosed EMF may also be common in the general population living in endemic regions.

## Heredity vs environment in DCM in Africa

DCM is a primary disorder of heart muscle that is characterised by dilatation and impaired contraction of the chambers of the heart. Presentation is usually with heart failure, which is progressive, with a four-year mortality of 34% after the onset of symptoms.^8^ DCM probably represents a final common expression of myocardial damage that could be provoked by multiple insults, including haemodynamic, infective, immunological, toxic, nutritional and genetic factors. It has been established that an intensive strategy of clinical investigation, which includes endomyocardial biopsy and coronary angiography, where indicated, yields a specific diagnosis in up to 50% of patients with previously unexplained DCM. 8

The cases of DCM that remain unexplained even after intensive investigation are a major challenge to the clinician and the researcher. The aetiological factors that have been examined in Africans include ‘burnt-out’, untreated hypertension, infection and myocarditis, auto-immune mechanisms, iron overload, excessive alcohol intake, nutritional deficiency, and pregnancy. Sliwa and her colleagues recently conducted a general overview of studies of these potential aetiological factors.^6^

A major advance in the study of the pathogenesis of unexplained DCM in other parts of the world has been the demonstration that 20 to 50% of cases are familial, suggesting that genetic factors may be involved in the aetiology of the condition.9 Reported families most commonly are compatible with autosomal-dominant inheritance, but some with X-linked and autosomal-recessive inheritance have been documented. Familial DCM is caused by mutations in at least 25 chromosome loci where genes encoding contractile, cytoskeletal and calcium regulatory proteins have been identified, underlining the genetic heterogeneity of the condition.^10^

To the best of our knowledge, the first report of familial DCM in Africa described twin brothers in Uganda.^11^ Brink subsequently documented a condition characterised by hereditary dysrhythmic congestive cardiomyopathy,^12^ and Przybojewski described two brothers of Afrikaner ancestry from South Africa with idiopathic DCM.^13^ More recently, Fernandez *et al.* have shown that familial progressive heart block type II, which was initially reported in 1977, may be associated with DCM in the late stages of the disease.^14^,^15^

Apart from these reports, we are not aware of systematic family studies that have been conducted to establish the frequency of familial DCM in Africans, such as has been done elsewhere.^16^ Nevertheless, several gene association studies have been conducted, which suggest that heredity may play a role in the susceptibility of Africans to DCM.

An association with HLA-DR1 and DRw10 antigens has been reported in South African patients, implying that genetically determined immune-response factors play a role in the pathogenesis of some individuals with DCM.^17^ A common mitochondrial DNA polymorphism (T16189C) has also been found to be a genetic risk factor for DCM in a South African cohort, with a population-attributable risk of 6%.^18^ These genetic associations have been replicated in other populations, suggesting that they are likely to represent genuine genetic risk factors for DCM worldwide.^6^ Mutation screening studies in patients with idiopathic and familial DCM have identified a family with early-onset DCM caused by a known mutation in the troponin T gene (Arg141Trp), but failed to reveal mutations in the cardiac and skeletal actin genes.^19^,^20^

## HIV infection: an environmental trigger for DCM?

The advent of the human immunodeficiency virus (HIV) epidemic in Africa presents new opportunities for the study of the interaction between an environmental factor, such as HIV, and heart muscle disease.^21^ It has long been hypothesised that idiopathic DCM, which is endemic in Africa, may represent ‘burnt-out’ viral myocarditis.^6^ It is known that about 15% of patients with proven viral myocarditis progress in later life to DCM which is indistinguishable from idiopathic DCM on clinical, virological and histological grounds.^6^ The association of HIV infection with cardiomyopathy was recognised in the early stages of the HIV epidemic.^22^ Although the percentage of people living with HIV who develop clinically apparent cardiomyopathy is relatively small, the disease burden could be substantial in the face of the exceptionally high prevalence of HIV infection in sub-Saharan Africa.^21^

Cross-sectional and retrospective studies suggest that cardiomyopathy is the leading cause of heart disease, among other systemic and protean manifestations of immunosuppression, in acutely ill hospitalised patients with HIV in Africa. While some patients with left ventricular dysfunction present with features of cardiac failure, the majority has left ventricular dysfunction detected only by echocardiography, without any clinical suggestion of heart failure. HIV-associated cardiomyopathy is characterised by global systolic functional impairment with or without left ventricular dilatation.

In studies of asymptomatic patients with HIV infection, left ventricular systolic dysfunction is found less frequently. In a study of 49 asymptomatic patients, Longo-Mbenza *et al.* found no evidence of systolic dysfunction but a high frequency of diastolic dysfunction (86%) and left ventricular hypertrophy (47%).^23^ However, in other studies, the prevalence of dilated cardiomyopathy in non-hospitalised ambulant HIV-positive patients may be as high as 28%, suggesting that Africans with HIV infection may be more susceptible to cardiomyopathy than their counterparts in the West, where prevalence rates of 15% have been reported.^21^ The association of cardiomyopathy with more advanced immunosuppression and lower CD4 counts, which was found in the African series, is consistent with international experience.

## Heredity vs environment in EMF

**Fig. 1.**
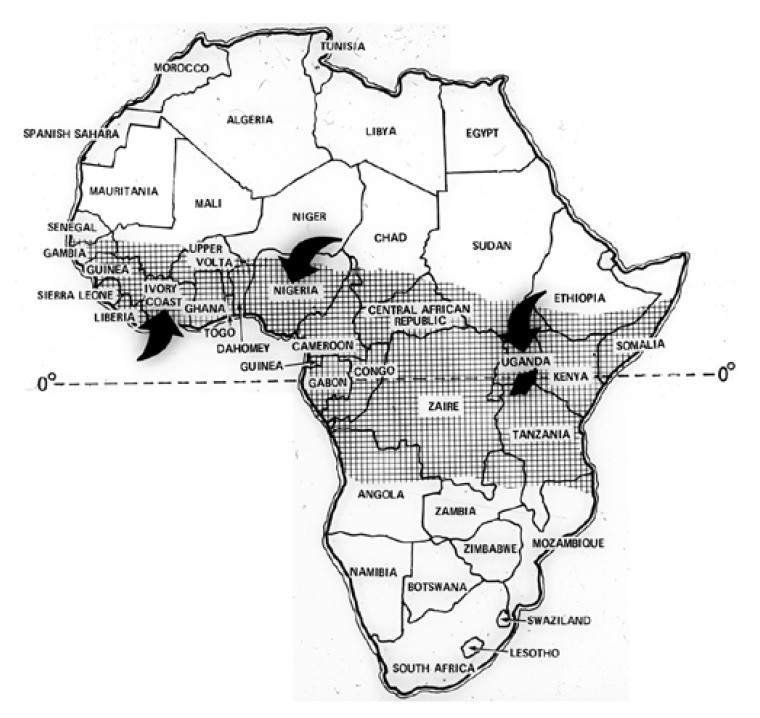
The geographic distribution of endomyocardial fibrosis in Africa is shown in the shaded area. Arrows indicate the location of major centres of research in this disease.

Endomyocardial fibrosis is a form of restrictive cardiomyopathy in which dense fibrosis in the mural endocardium restricts ventricular diastole and entraps the papillary muscles of the atrioventricular valves. EMF occurs mainly in tropical and subtropical areas worldwide. In Africa, the first awareness of EMF is ascribable to Williams in Uganda.^24^ The disorder was named endomyocardial fibrosis in a subsequent clinicopathological study,^25^ and was given the eponym, Davies’ disease^26^,^27^ in deference to the seminal contributions of Davies.^28^ EMF has been widely reported in Uganda, Kenya, Tanzania, Mozambique, Gabon, Congo, Cameroon, Sudan, Nigeria, Coté d’Ivoire and Ghana (Fig. 1). It is uncommon in northern and southern Africa. EMF in Africa is more commonly right sided or bilateral, and rarely left sided.

EMF is said to be the most common form of heart disease in Ugandan hospitals where it accounts for nearly 20% of cases referred to an echocardiography service.^29^ The only epidemiological survey performed in Africa to the best of our knowledge was based on an echocardiographic diagnosis of EMF in the Inharrime district of Mozambique. In a sample of 948 inhabitants from an endemic area and aged between four and 45 years, a prevalence of 8.9% was reported, suggesting that EMF was a major form of heart disease in the region (B Ferreira, unpublished data, 2001). The disease predominates in children and young adults, with a peak incidence in the ages of 11 and 15 in both sexes.

**Fig. 2.**
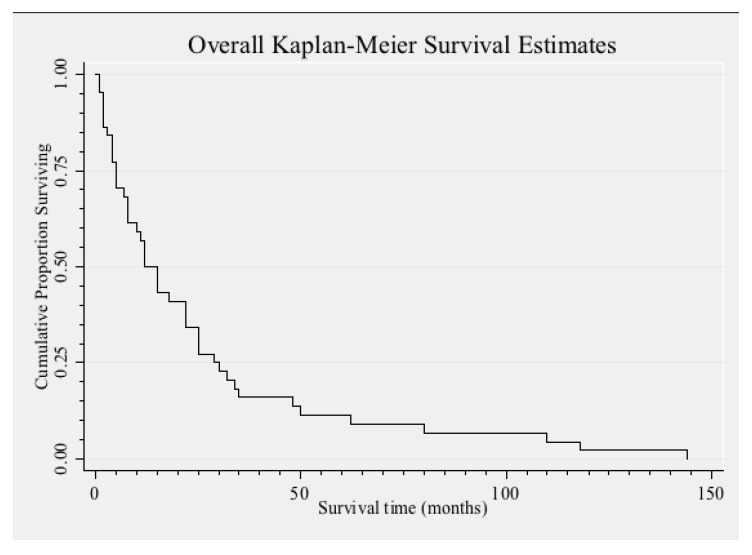
Survival of 46 necropsy cases of EMF patients after first symptoms.

The clinical features of EMF depend on the stage of disease and the anatomical involvement of the heart. Thirty to 50% of children and adolescents report an initial illness with fever, chills, night sweats, facial swelling and urticaria.^30^ Retrospective history of fever in malaria-prone areas is a confounding fact. In natural history, there may be rapidly developing cardiac failure and early death, or evolution to established and apparently inactive EMF with predominantly right ventricular or left ventricular disease. Ascites with little or no peripheral oedema is the characteristic clinical feature of end-stage EMF, regardless of which ventricle is involved. Unlike congestive right heart failure, the ascites is an exudate in 75% of cases and is associated with peritoneal fibrosis.^31^ An exudative pericardial effusion of variable degree is a common presentation. Prognosis is poor in this condition and death usually occurs within two years of diagnosis. Response to conservative treatment for heart failure is poor with a 75% mortality rate at two years (Fig. 2).^32^

Several environmental (geography, social deprivation, infection, eosinophilia) and inherited (ethnicity) factors have been implicated in the pathogenesis of EMF in Africa. There is, however, no compelling evidence supporting the contribution of inherited factors in the pathogenesis of EMF. While in Uganda the disease is said to be more common among immigrants from neighbouring Rwanda and Burundi who have settled in specific geographic districts of the country,^26^,^27^,^33^ EMF also occurs in other Ugandans and in foreigners who have lived in tropical Africa,^34^,^35^ suggesting that the disease cannot be explained solely on the basis of ethnic factors or social deprivation. Apart from a few isolated reports of familial cases of EMF in endemic regions,^36^,^37^ the role of familial and genetic factors has not been studied systematically in this condition.^6^

Eosinophilia has also been proposed as a major risk factor for EMF in both Uganda and Nigeria.^33^,^38^ The level of eosinophilia has been found to be inversely related to the duration of illness, leading to the notion that patients who do not have eosinophilia are at a late stage of EMF. The usual presentation of EMF is in end-stage restrictive cardiomyopathy. There has been a suggestion that Loeffler’s endocarditis, which is occasionally seen in non-tropical countries, and EMF, represent the extremes of the same disease. Eosinophils contain major basic protein, cationic protein, protein X and other substances which are released during degranulation and which are thought to be toxic to the endo- and myocardium, resulting in mural thrombosis and fibrosis.^39^

The pathogenesis of EMF remains elusive. It appears, however, that the conditioning factor is geography (in its broadest sense, to include climate and socio-economic conditions), the triggering factor may be an as yet unidentified infective agent, and the perpetuating factor may be eosinophilia. The demonstrated role of auto-immunity requires further study.^40^ The role of heredity remains to be established through, for example, genetic epidemiological studies of twins, families and adoptees.^41^

## Hypertrophic cardiomyopathy

Hypertrophic cardiomyopathy (HCM) is recognised in Africans and other populations as an autosomal-dominant disorder that is caused by mutations in at least 11 different genes that code for sarcomeric proteins.^6^ The majority of HCM-causing mutations have arisen independently in most families studied, suggesting that the majority occurred relatively recently as new mutations. This finding predicts that HCM is likely to be evenly distributed among different populations worldwide.^42^ Experience elsewhere in the world has revealed numerous mutations in the sarcomeric protein genes, such that many families have a ‘private’ mutation. In South Africa, however, there are three founder mutations that recur in 45% of genotyped patients of European and mixed ancestry.^43^ Consequently, South African patients with HCM referred for molecular diagnosis are initially screened for the three founder mutations (ie, -MHC Arg403Trp, -MHC Ala797Thr and cTNT Arg92Trp), and more extensive screening is performed only in their absence.^9^

## Arrhythmogenic right ventricular cardiomyopathy

Arrhythmogenic right ventricular cardiomyopathy (ARVC) is characterised macroscopically by dilatation and reduced systolic function of the right ventricle, and microscopically by myocardial cell loss with partial or total replacement of the right ventricular muscle by adipose and fibrous tissue. ARVC was reported for the first time in Africa in 2000,^44^ about 40 years after the first case was described by Dalla Volta in 1961.^45^ The disease is known to be familial in about half of affected patients. Initial information in South Africa suggests that ARVC occurs in all segments of the population, and that its clinical features, frequency of familial disease and outcome are similar to evidence that has been gathered elsewhere in the world.^6^

## Conclusion

**Fig. 3.**
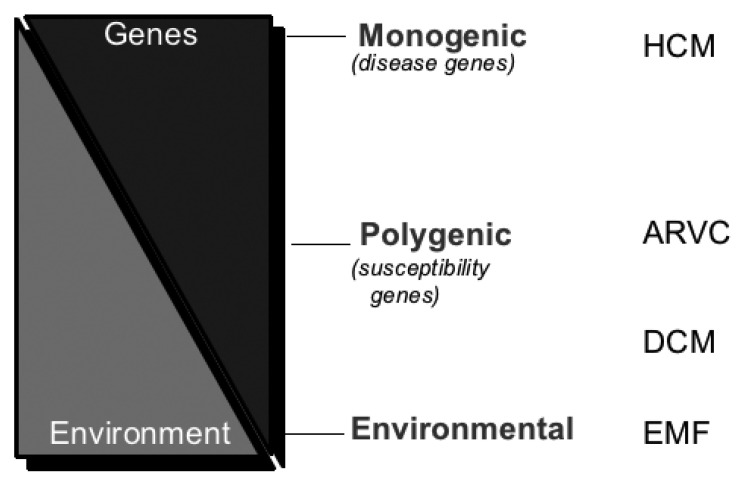
Genetic and environmental contribution to cardiomyopathy occurs along a continuum. HCM, hypertrophic cardiomyopathy; ARVC, arrhythmogenic right ventricular cardiomyopathy; DCM, dilated cardiomyopathy; EMF, endomyocardial fibrosis. (Courtesy of Prof H Watkins, University of Oxford.)

The contribution of inherited factors to the pathogenesis of the cardiomyopathies vary from hypertrophic cardiomyopathy (almost always genetic) to EMF (probably wholly environmental) (Fig. 3). Much work remains to be done to discover the size and nature of genetic and environmental contributions to various forms of DCM and EMF, which are endemic in Africa.
